# Organisational capacity and its relationship to research use in six Australian health policy agencies

**DOI:** 10.1371/journal.pone.0192528

**Published:** 2018-03-07

**Authors:** Steve R. Makkar, Abby Haynes, Anna Williamson, Sally Redman

**Affiliations:** 1 The Sax Institute, Ultimo, New South Wales, Australia; 2 Sydney School of Public Health, University of Sydney, Camperdown, New South Wales, Australia; Universidad de las Palmas de Gran Canaria, SPAIN

## Abstract

There are calls for policymakers to make greater use of research when formulating policies. Therefore, it is important that policy organisations have a range of tools and systems to support their staff in using research in their work. The aim of the present study was to measure the extent to which a range of tools and systems to support research use were available within six Australian agencies with a role in health policy, and examine whether this was related to the extent of engagement with, and use of research in policymaking by their staff. The presence of relevant systems and tools was assessed via a structured interview called ORACLe which is conducted with a senior executive from the agency. To measure research use, four policymakers from each agency undertook a structured interview called SAGE, which assesses and scores the extent to which policymakers engaged with (i.e., searched for, appraised, and generated) research, and used research in the development of a specific policy document. The results showed that all agencies had at least a moderate range of tools and systems in place, in particular policy development processes; resources to access and use research (such as journals, databases, libraries, and access to research experts); processes to generate new research; and mechanisms to establish relationships with researchers. Agencies were less likely, however, to provide research training for staff and leaders, or to have evidence-based processes for evaluating existing policies. For the majority of agencies, the availability of tools and systems was related to the extent to which policymakers engaged with, and used research when developing policy documents. However, some agencies did not display this relationship, suggesting that other factors, namely the organisation’s culture towards research use, must also be considered.

## Introduction

There have been increased calls worldwide for policymakers to make greater use of research when formulating and implementing health policies in order to promote more sustainable and equitable health spending and use of resources, minimise health inequities, and improve health outcomes on a global scale [1–8]. Unfortunately, the use of research in health policymaking is less than optimal [4, 5, 9–16].

Evidence indicates that the suboptimal use of research to inform health policy is due to numerous barriers. Firstly, policymakers argue that research is often not presented in a clear, user-friendly, and summarised format with clear policy implications [5, 16–22]. Secondly, policymakers report deficiencies in skills and confidence in acquiring, appraising, interpreting, synthesising, and applying research to policy [14, 17, 19, 21, 22]. Infrastructural support for these actions (e.g., journal subscriptions; skills training; research support staff such as librarians, knowledge brokers or consultants) has also been reported as insufficient by policymakers [8, 17–20, 23, 24]. Finally, policymakers and researchers have described a lack of collaborative research, despite evidence demonstrating this to be an important facilitator of research dissemination and uptake [20, 25–27].

**Fig 1:**
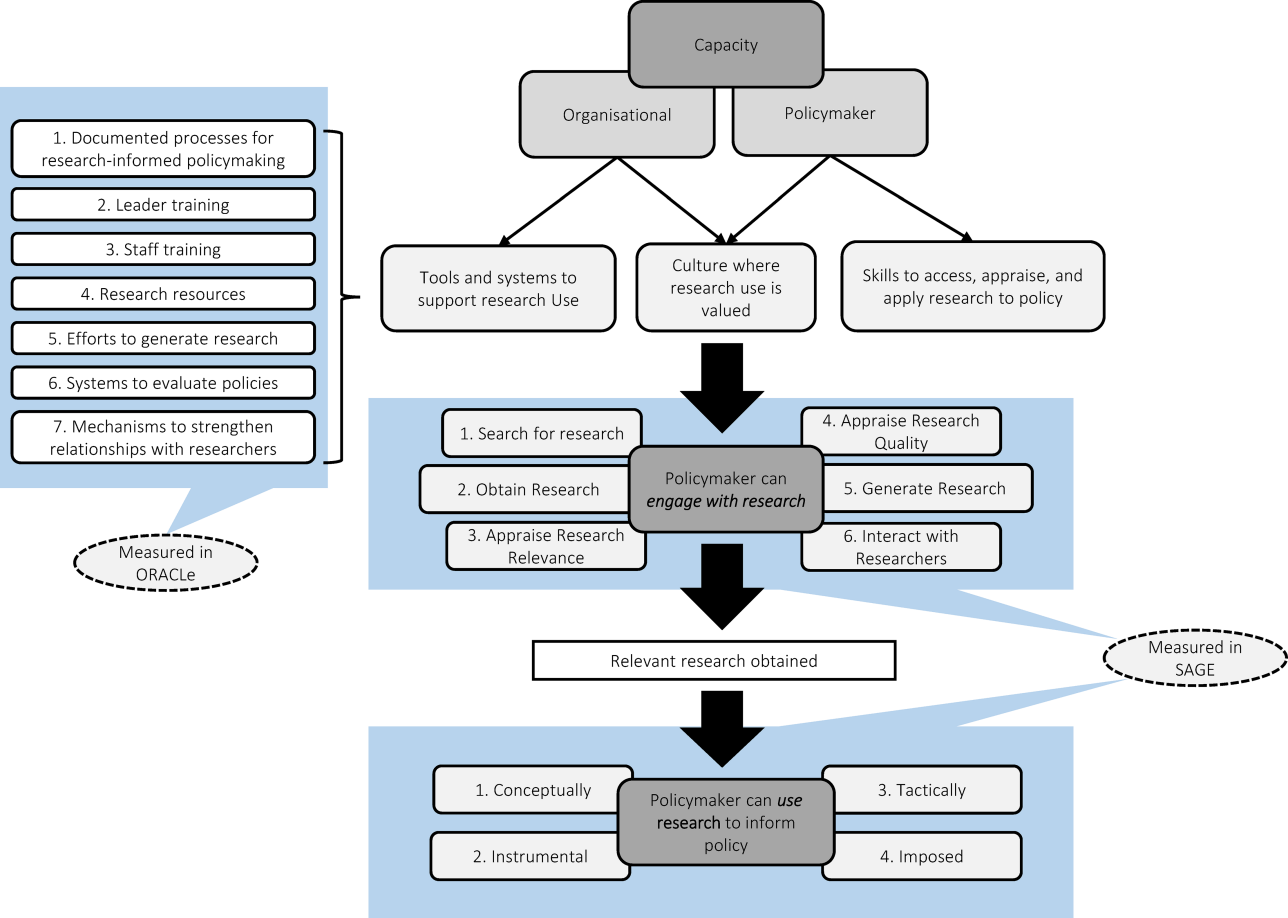
The influence of organisational and policymaker capacity on policymakers’ engagement with, and use of research in health policymaking. Based on the SPIRIT Action Framework as described in: Redman, S., Turner, T., Davies, H., Williamson, A., Haynes, A., Brennan, S.,… Green, S. (2015). The SPIRIT Action Framework: A structured approach to selecting and testing strategies to increase the use of research in policy. Soc Sci Med, 136–137, 147–155. doi: 10.1016/j.socscimed.2015.05.009.

These barriers could potentially be overcome if organisations and policymakers have a sufficient level of *capacity* to enable greater use of research in policy. According to the SPIRIT Action Framework [28], capacity encompasses three main aspects: (a) a culture where research use in policymaking is valued by the organisation and staff; (b) sufficient skills and knowledge among staff to access, appraise, and apply research to policy; and (c) tools and systems available to support research use by staff and to establish collaborative relationships with researchers (see Fig 1).

The framework emphasises that where there is sufficient capacity, policymakers can initiate six specific research engagement actions that include (i) searching for, and (ii) obtaining research; (iii) appraising its relevance to the policy issue and (iv) appraising its quality or scientific rigour; (v) generating new research and/or data analyses (especially when relevant research is not available); and (vi) interacting and collaborating with researchers. Once relevant research has been accessed and/or generated, it can be used to inform policymaking in four ways. Specifically, research may: (i) directly inform decisions relating to the identified policy issue(s) (instrumental use) [29, 30], (ii) clarify understanding about the policy issue without directly influencing the decision (conceptual use)[13, 31, 32], (iii) justify and/or persuade others to support a predetermined decision (tactical use)[30, 33], or (iv) be used to meet legislative, funding, or organisational requirements (imposed use)[31]. The framework predicts that using research to *inform* policies will ultimately lead to better health services and outcomes.

Detailed and systematic measurement of organisations’ current research use capacity is essential to identify the tools and systems needed to drive improvement in policymakers’ use of research [34]. Previous research has shown that there are four broad types of capacity-building initiatives that can support evidence-informed policymaking in organisations [35]. These initiatives included (i) availability of staff with expertise to promote and support research use [19, 35]; (ii) mechanisms to establish links with researchers and opinion leaders outside the organisation [6, 7, 18, 19, 21, 22, 35–40]; (iii) technical infrastructure to facilitate research access (e.g., journal and database subscriptions; knowledge management systems for efficient storage and retrieval of research findings) [8, 35, 41–43]; and (iv) training programs to improve staff skills in accessing, appraising and applying research to policy [4, 6, 35, 44, 45] and to build agency executives’ skills and capacity for promoting research use within their organisations [35, 46]. Only a few studies have systematically investigated the availability of these tools, systems, and processes within policy agencies to support research use (e.g., [35, 45, 47]. Most of the research has instead focused on documenting *barriers* and *facilitators* to research use beyond organisational factors such as political forces, stakeholder opinions, or the availability of relevant, trustworthy, and timely research [6, 27, 48–51]). Furthermore, studies have yet to examine whether the availability of these tools and systems within agencies is related to policymakers’ actual engagement with, and use of research in policymaking. This information is likely to be useful in identifying organisational tools and systems that have the greatest potential for building health policymakers’ capacity to use research in policymaking.

The aim of the present study was to undertake a systematic investigation of the tools and systems available to support the use of research in health policymaking within Australian agencies located in Sydney, New South Wales. We also investigated whether the availability of these tools and systems was related to the extent to which policymakers engaged with and used research when developing policies.

## Materials and methods

### Agencies

Six agencies with a role in health policy based in Sydney, New South Wales, Australia participated in the current study: five state government and one national organisation. As part of the recruitment criteria an agency was eligible to participate if (a) a significant proportion of its work was in health policy or program development, and (b) there were at least 20 staff involved in health policy or program development or evaluation. For the purposes of this paper, we refer to such staff as *policymakers–*individuals employed in policy agencies, who draft or write health policy documents, develop health programs, or make significant contributions to policy decisions relating to health services, programs, and/or resourcing [52]. Three of the participating agencies concentrated on specific areas of health (e.g. cancer), and three worked more broadly across public health and health systems improvement. Of the five state government agencies, four were board-governed statutory organisations that co-reported to the NSW Minstry of Health, and the fifth was a division within the Ministry itself. The national agency reported to a board but was entirely funded by the Federal Department of Health. Thus all were government funded and subject to fluctuations in state and federal budgets. All had been operating for at least three years but were subject to recent or current restructures.

### Measures

#### Organisational tools and systems

To measure agencies’ tools and systems to support research use, we used the Organisational Research Access, Culture and Learning (ORACLe) measure [34] developed by the Centre for Informing Policy in Health with Evidence from Research (CIPHER) at the Sax Institute, Sydney, Australia. ORACLe consists of two components: (a) a semi-structured interview, and (b) a scoring guide. The interview comprises 23 questions that capture information about the extent to which a range of tools and systems to support research use are available within an organisation, with a focus on initiatives that have been developed in the six months prior to measurement. The questions cover seven key domains of organisational capacity to support research use as displayed in Table 1. Raters use the scoring guide to score each question on the following three-point scale: 1 = the tool or system is not present, 2 = it is present to a limited/moderate extent or 3 = it is present to a large extent. Questions under the same domain are averaged, yielding seven domain scores, each out of 3. These domain scores are entered into an empirically derived algorithm to produce an overall capacity score out of 3 for that organisation (where where 0–0.99 = limited, 1–1.99 = moderate, and 2–3 = extensive overall capacity) [34]. This algorithm gives greater weight to domains that have greater relative importance for building organisational capacity, based on discrete choice modelling of surveys completed by knowledge translation experts. More information on how ORACLe is scored and how the scoring algorithm was derived from a discrete choice consultation with international experts can be found elsewhere [34]. In the present study, SM and AW used the marking guide to score ORACLe interviews from each of the six participating agencies.

**Table 1:** Purpose, components, constructs, and domains assessed by each of the key outcome measured used in the present study.

Measure	Purpose	Components	Key constructs assessed	Domains Assessed
ORACLe (Organisational Research Access, Culture, and Learning)	To document and score the availability of systems and tools within the organisation that support the use of research in policy by staff	Structured interview and marking guide	Organisational tools and systems to support research use in policy	1. Documented processes to develop policy that encourage or mandate the use of research 2. Tools and programs to assist leaders of the organisation to actively support the use of research in policy development 3. Programs to provide staff with training on how to use research in policy 4. Availability of supports and tools to help staff access and apply research findings 5. Systems and methods to generate new research to inform the organisation’s work 6. Methods to ensure adequate evidence-informed evaluations of the organisations’ policies 7. Mechanisms that help strengthen staff relationships with researchers.
SAGE (Staff Assessment of enGagement with Evidence)	To document and score policymakers’ engagement with, and use of research in the development of discrete policies and programs	Structured interview and scoring tool/checklist	Research Engagement Actions	1. Strategies to search for research 2. Types of research obtained 3. Strategies to appraise the relevance of research obtained 4. Strategies to appraise the quality of research obtained 5. Efforts to generate new research or analyses 6. Efforts to interact with researchers
			Research Use Actions	7. Conceptual research use 8. Instrumental research use 9. Tactical research use 10. Imposed research use

#### Research engagement and use

The current study also utilises a newly developed measure called SAGE (Staff Assessment of enGagement with Evidence), which assesses ten domains—six research engagement actions and four research use actions—in relation to a specific policy document completed in the last six months (see Table 1 and Fig 1). SAGE consists of a semi-structured interview with the person most heavily involved in producing the document and a scoring checklist. In the interview, policymakers are asked: (i) whether or not research was used to inform the document; (ii) what *research engagement actions* were undertaken; (iii) how research was used; and (iv) what barriers and facilitators impacted on their use of research (see Table 1). Interviews were audio recorded and professionally transcribed. The interviewer checked, corrected, and de-identified transcripts before they were scored.

The scoring checklist breaks down each of the measured domains into their key *subactions*. Subactions are the essential features/actions of each domain. For example, a key subaction for *Searching for Research* is looking through academic literature databases such as Medline. If a particular subaction was performed by the policymaker on the basis of his or her interview, it is ticked off in the scoring checklist. Each subaction has a different point value assigned to it based on its importance in facilitating evidence-informed health policymaking. The degree of importance of each subaction was established through conjoint analysis of surveys completed by over 50 local and international experts in knowledge translation [53, 54]. The points for all ticked subactions are summed to give a score of 0–9 for that particular domain (where 0–2.99 indicates limited, 3–5.99 moderate, and 6–9 extensive efforts to engage with, or use research). Full information on scoring SAGE and the complete tool is available elsewhere [53–56]. The reliability and validity of SAGE has also been demonstrated [55]. In this study, author SM applied the SAGE scoring checklist to score the interview transcripts on the ten measured domains.

### Data collection

Data was collected as part of SPIRIT (Supporting Policy In health with Research: an Intervention Trial) [57], a stepped wedge intervention trial designed to improve the capacity of agencies and staff to engage with and apply research in policy work [28, 57]. Prior to the commencement of SPIRIT, the agencies completed a suite of measures including ORACLe and SAGE, in order to evaluate their level of capacity and research use respectively. Information about the agencies and how they were recruited is described elsewhere [57]. For ORACLe, a senior staff member from each of the six agencies (labelled A1 to A6 in this study) was nominated to undertake the ORACLe interview. Senior staff members (executive staff and CEOs) were targeted since they were most likely to possess accurate knowledge of the systems, tools, and structures to support research use within their organisations. Each of the six participating agencies also selected four policy documents which, in their view, best represented the use of research in their work. These documents were used as a proxy for agencies’ wider policy development processes and were the focus of SAGE interviews. The policymaker identified as having contributed most to each document’s development was invited to participate in the SAGE interview. Four interviews were completed for each of the six agencies (24 interviews altogether). Both the ORACLe and SAGE interviews were conducted by a single interviewer, by phone (except when the interviewee requested a face-to-face interview) and ranged from 30–60 min in length.

### Data analysis

ORACLe and SAGE scores were calculated for each agency. For ORACLe, each agency received a score out of 3 for each of the seven capacity domains measured by the tool. These domain scores were entered into the algorithm to obtain a score out of 3 indicating the agency’s overall capacity to support research use in policymaking. For SAGE, each of the four policy documents were scored on the 10 domains measured by the tool: six research engagement actions and four research use actions (each scored out of 9), and the scores across the four documents were averaged. To evaluate the relationship between capacity and research engagement/use, associations between ORACLe and SAGE scores were examined qualitatively, since the sample was too small to calculate correlation coefficients.

### Ethics

Ethics approval was granted by the University of Western Sydney Human Research Ethics Committee HREC, approval H10440. Written informed consent was obtained from all interviewees.

## Results

### Organisational tools and systems

Data describing the systems and tools within each agency are displayed in Table 2 and elaborated upon below. Data are organised under the seven domains of systems and tools to support research use in policy as assessed in ORACLe (see Table 1). All data regarding the agency’s systems and tools derived from the reports of a single executive per agency.

**Table 2:** Systems and tools to support research use within each agency in the six month period prior to measurement.

	1. Documented processes to develop policy that encourage or mandate the use of research	2. Tools and programs to assist leaders actively support the use of research in policy development	3. Programs to provide staff with training on how to use research in policy	4. Supports and tools available to help staff access and apply research findings	5. Efforts to generate new research	6. Processes to enable evidence-informed evaluations of the organisations’ policies	7. Mechanisms that help strengthen staff relationships with researchers
Agency 1	Standard written guidelines available with explicit requirement to use research.	No programs for leaders. Performance management implies research use expertise. Irregular* reference to research in internal communications.	No training programs for staff.	Internal dissemination of research less than twice a month. Access to experts to provide research support. Access to libraries, and most relevant journal subscriptions and databases. Standard processes for commissioning reviews of research. Centralised system for storing research knowledge but not well-organised.	Several external research projects undertaken.	Explicit requirement to evaluate policies, but no documented processes to inform evaluation.	Regular attendance at conferences and involvement of researchers in advisory committees. Several contractual and one informal relationship with external research organisations.
Agency 2	Written guidelines available, but not detailed, and research use is implied	Programs open to all staff including leaders. Performance management implies research use expertise. Internal communications regularly refer to research (at least once a month).	Staff training provided on a needs basis. Participation in training considered in performance management for all staff.	Frequent** internal dissemination of research. Access to libraries, and most relevant journal subscriptions and databases. Reference management software available. Ad-hoc, situation-dependent methods to commission research reviews. Centralised system for storing research knowledge but not well-organised.	Several internal research projects undertaken. One external project undertaken.	Explicit requirement to evaluate policies, but no documented processes to inform evaluation.	Regular attendance at conferences and involvement of researchers in advisory committees. Several formal (contractual) and informal relationships with research organisations. Several staff hold adjunct appointments in research organisations.
Agency 3	Standard written guidelines available, but research use implied	No programs for leaders. Performance management makes no reference to research use expertise. Internal communications do not refer to research.	Regular staff training opportunities provided. Participation in training considered in performance management for all staff.	Frequent** internal dissemination of research. Resources to help staff appraise research. Access to experts to provide research support. Access to libraries, and most relevant journal subscriptions and databases. Reference management software available. Standard processes for commissioning reviews of research. Well-organised/structured systems for storing and managing research knowledge.	Several internal research projects undertaken.	Explicit requirement to evaluate policies, and standardised documented processes available, but do not explicitly require research use.	Regular attendance at conferences and involvement of researchers in advisory committees. Several formal (contractual) and informal relationships with research organisations.
Agency 4	No written guidelines or documentation	Programs open to all staff including leaders. Performance management makes no reference to research use expertise. Irregular* reference to research in internal communications.	No training programs for staff.	Frequent** internal dissemination of research. Access to experts to provide research support. Access to libraries, and most relevant journal subscriptions. Some database access. Reference management software available. Standard processes for commissioning reviews of research. Centralised system for storing research knowledge but not well-organised.	Several internal research projects undertaken.	Evaluation of policies is expected (not required). No documented evaluation processes.	Regular attendance at conferences and involvement of researchers in advisory committees. Several formal (contractual) and informal relationships with research organisations. Several staff hold adjunct appointments in research organisations.
Agency 5	Yes, standard written guidelines, but research use implied	No programs for leaders. Performance management makes no reference to research use expertise. Irregular* reference to research in internal communications.	Staff training provided on a needs basis. Participation in training considered in performance management of select staff.	Frequent** internal dissemination of research. Access to experts to provide research support. Access to some relevant journal subscriptions only. Reference management software available. Standard processes for commissioning reviews of research. Centralised system for storing research knowledge but not well-organised.	Several internal and external research projects undertaken.	Explicit requirement to evaluate policies. Evaluation processes available but not standardised across programs, nor explicitly require research use.	Regular attendance at conferences and involvement of researchers in advisory committees. Several formal (contractual) and informal relationships with research organisations. Several staff hold adjunct appointments in research organisations.
Agency 6	Yes, standard written guidelines, but research use implied	No programs for leaders. Performance management makes no reference to research use expertise. Irregular* reference to research in internal communications.	No training programs for staff.	Access to experts to provide research support. Access to libraries, some journal subscriptions and most relevant databases. Ad-hoc, situation-dependent methods to commission research reviews. No dissemination of research or centralised research storage system.	Multiple internal and external research projects undertaken.	Explicit requirement to evaluate policies, but no documented processes to inform evaluation.	Regular attendance at conferences. No involvement of researchers in advisory committees. Several contractual and one informal relationship with external research organisations.

Note

*Irregular = less than once per month

**Frequent = several times per month

#### Processes for developing policy that mandate or encourage use of research

In four of the six agencies (A1, A3, A5, and A6), explicit, detailed documented processes for how policies should be developed were available, which often took the form of *“a policy on how to write policies”*, or “*written documents and templates”*. A2 also had documented processes on how policies should be developed but they lacked detail. In A1, these documented processes explicitly required consultation of research findings or researchers; whereas in A3, A5, and A6, the requirement to use research was not directly stated. In A4, such documented policy development processes were still in the process of being established.

#### Programs and systems to assist leaders in supporting the use of research in policy

No agency had training programs focused on building leaders’ confidence and skills in using research to inform policy. A2 and A4 did offer training to all staff including leaders, but this did not address leadership/managerial issues, and was not *“specifically targeted [at] research”*. Furthermore, none of the performance management systems for leaders in the agencies studied covered expertise in research use. Finally, in all agencies except A3, systems to enable internal communications from leaders to staff (e.g., email updates, newsletters, blogs, tweets, bulletins) were in place. References to the latest research in these communications occurred on a weekly basis within A1, whereas in the remaining agencies they were either *“ad-hoc”* or infrequent (e.g., quarterly).

#### Programs to provide staff with training on how to use research in policy

A2, A3, and A5 participants reported that their staff were provided with access to training programs to improve research skills. This included external professional development courses (e.g., on using EndNote), *“internal workshops on critical appraisal”* or using research databases, and *“external people coming in [and] doing formal training”*. In A3 these programs were offered consistently, whereas within A2 and A5, they were only offered if staff *“identified that there’s a need for them”* to participate in such training programs. In A2 and A3, participation in training was considered in staff performance management, whereas in A5, it was only considered if it *“came up as an issue”* for the employee.

#### Systems and tools and help staff access and use available research

Within each agency, there were numerous systems to help staff access and obtain available research efficiently. For example, all agencies except A6 disseminated relevant and timely research to staff through various mechanisms such as email, social media, list servers, Google alerts, and journal clubs. In A2-A5 this occurred several times per month, and in A1 less than twice per month. A3 utilised an organised and indexed *“library management system”* for archiving this research and other knowledge from research. This system provided an efficient and effective means of sharing, editing, storing and retrieving research findings by staff. The remaining agencies (except A6) had centralised repositories for storing and retrieving research, but these were not indexed and therefore not easily searchable. Finally, four agencies (A1, A3, A4 and A5) reported having established systems for commissioning research reviews. These included specific research arms within the agency that conducted formative research (A3), and established relationships with centres that conduct rapid reviews (A1, A4, A5). The remaining agencies used *“very ad-hoc methods”* for commissioning reviews, which depended on *“what the project was about and how much [it costed]”*.

Participants reported that agencies provided numerous tools and supports to staff for accessing research. These included subscriptions to databases and relevant research journals, access to physical and/or electronic libraries, licenses for reference management software such as EndNote, and easy access to research expertise in the form of librarians, medical writing teams, research and evaluation teams, epidemiologists, medical staff, and scientists. Finally, A3 also provided staff with a *“critical appraisal tool”* that provided detailed, documented guidelines on appraising research. Such guidelines weren’t available in the other agencies.

#### Efforts to generate new research

All agencies except A1 reported conducting several research projects internally to generate new data to inform programs under development. These were conducted by research divisions (A3 and A5) or policy teams (A2, A4, A5) within the agency often in partnership with research organisations (A4, A5). Three agencies also reported commissioning research externally either via research partner organisations (A1) or external institutes and consultants (A4, A5).

#### Processes to enable research-informed policy evaluations

Although respondents from each agency (excluding A4) emphasised that policy and program evaluation was *“mandatory”* or *“an expectation and requirement”*, there was an absence of standardised documentation or procedural guidelines that specified how policies should be evaluated, except within A3. In this agency, program evaluations were guided by *“an evaluation framework”* which acted as “*a critical thinking tool… that defined… inputs [and] outputs… [and] how you would measure them”*. This framework, however, did not explicitly instruct staff to use research to guide the evaluation. Evaluation processes in A5 varied depending on the program being evaluated or the methods of evaluation staff within different divisions of the agency. These methods were loosely based on *“a program logic… [and] documentations [with] some core requirements”* and not standardised processes. Furthermore, A5 also commissioned consultants to conduct evaluations who utilised their own evaluation frameworks.

#### Mechanisms to help strengthen relationships with researchers

Participants reported that their agencies had multiple mechanisms to strengthen relationships with researchers. Firstly, each agency provided staff with opportunities to represent their organisation at numerous conferences and forums. Second, all agencies reported having established several contractual, and at least one informal, relationship with external research organisations (e.g., universities). Third, at least one staff member from three agencies (A2, A4, A5) was reported to hold an adjunct appointment with a university. Finally, in all agencies except A6, researchers were frequently invited to participate in policy advisory committees. These “*external advisors”* were reportedly sought out for their expertise and were said to be essential to the policy advisory process such that *“we wouldn’t set up such a mechanism without having them at the table”*.

### Relationship between organisational capacity to support research use and actual research engagement/use for each agency

**Fig 2:**
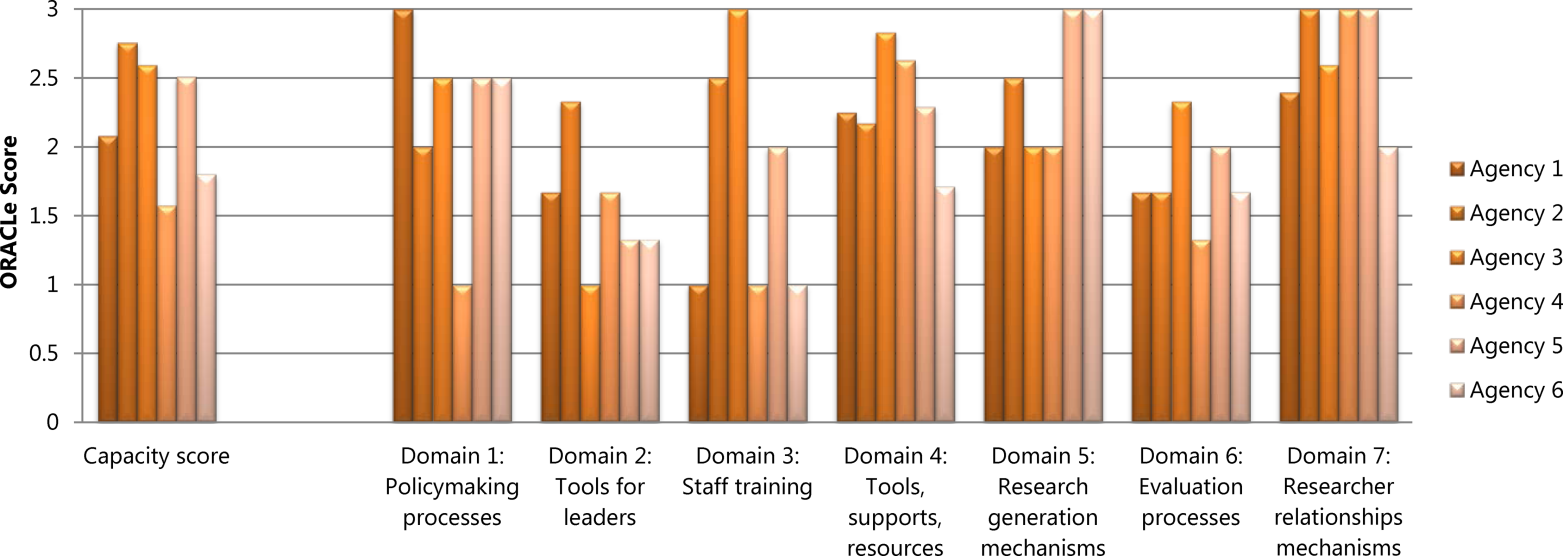
Agencies’ ORACLe scores for each capacity domain and total capacity scores.

**Fig 3:**
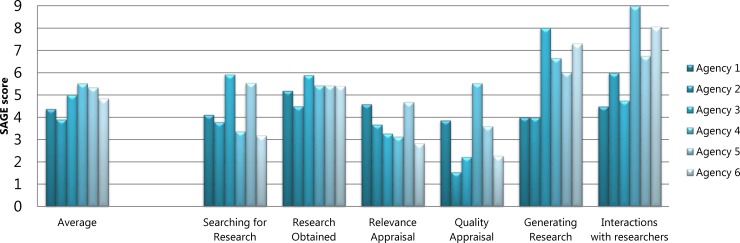
Research engagement actions scores. Agencies’ SAGE scores for individual research engagement actions and the average score for research engagement actions.

**Fig 4:**
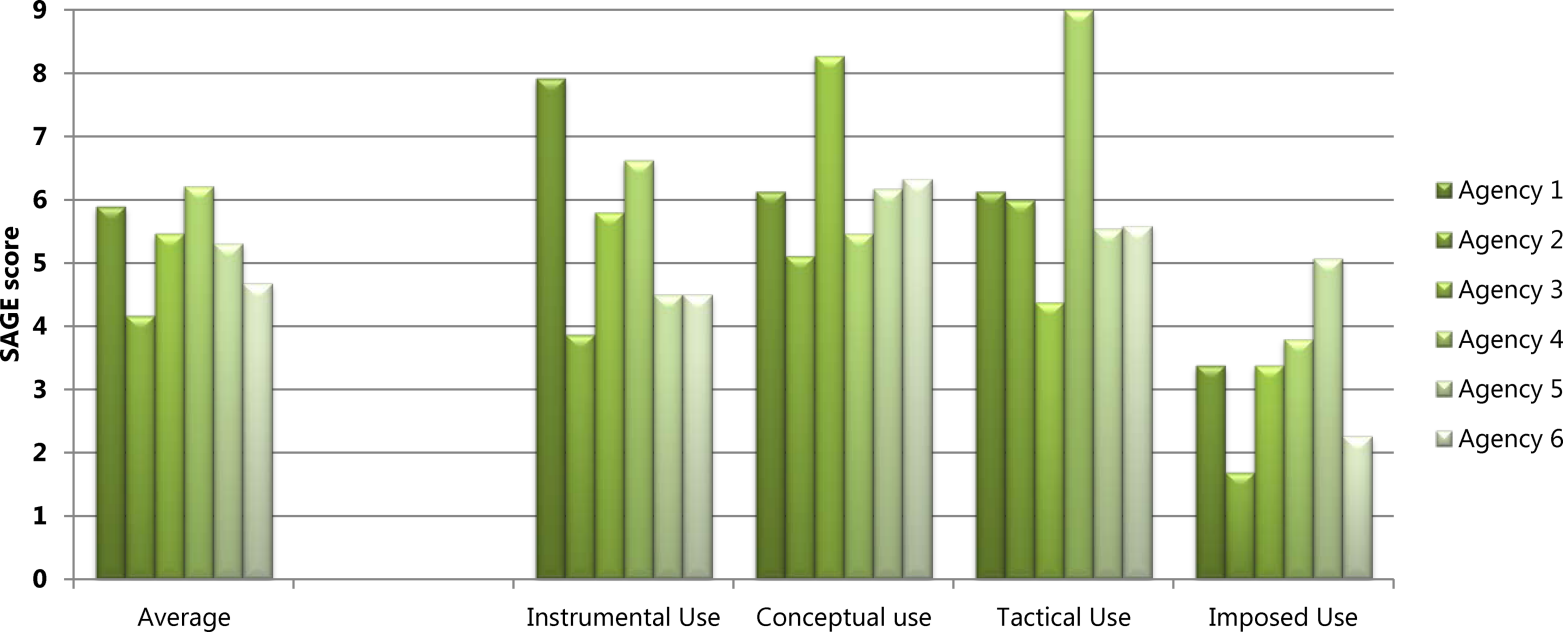
Research use scores. Agencies’ SAGE scores for each research use action and the average score for research use.

Fig 2 displays each agency’s scores on the seven ORACLe domains of systems and tools to support research use and the agency’s overall capacity score. Figs 3 and 4 show each agency’s SAGE scores for the six research engagement actions and four types of research use, respectively, as well as average scores for each. The results showed that for four out of the six agencies, ORACLe total scores (reflecting organisational capacity to support research use) were consistent with policymakers’ engagement with, and use of research as measured by SAGE.

A1, A3, and A5 had ORACLe scores reflecting extensive overall capacity to support research use and also displayed a relatively high degree of research engagement and/or use (Fig 2). A1’s high ORACLe scores were attributable to the agency having a documented guideline on writing policies, offering numerous resources to help staff access research (e.g., access to relevant journals, databases, a library and library staff; clear protocols for commissioning research reviews), and numerous mechanisms to strengthen relationships with researchers. In line with this, their SAGE data showed research was used instrumentally (i.e., used to directly inform policy priorities and/or alternatives) to an extensive degree (Fig 4). Policymakers from this agency also reported extensive conceptual and tactical research use.

A3’s overall ORACLe score also revealed extensive capacity to support research use, attributable to having clear frameworks on how to develop policy, ongoing training for staff to improve research skills, and a wide range of resources such as databases, journal subscriptions and guide documents to help staff use existing research (Fig 2). In line with this, their SAGE data suggested A3’s policymakers’ efforts to engage with research were in the moderate to extensive range, particularly with regard to efforts to search for research and the range of research obtained (Fig 3). A3 staff also reported extensive efforts to generate new research and displayed extensive conceptual research use, and moderate to extensive instrumental use (Fig 4).

A5 also received ORACLe scores indicative of extensive overall capacity to support research use (Fig 2). This high overall score was attributable to reportedly extensive documented processes for policy development and evaluation, resources to help staff access existing research (e.g., dissemination of research across the agency, access to research expertise, access to some relevant journals), and clear processes for commissioning research reviews. This agency had also actively undertaken internal and external research projects in the past six months, and reported strong mechanisms to establish relationships with researchers. In line with this, SAGE data revealed that efforts to generate new research and interact with researchers were extensive and that relevance appraisal efforts were in the moderate range (Fig 3). Furthermore, policymakers reported moderately high tactical and imposed research use, and extensive conceptual research use (Fig 4).

A6 received a relatively low overall capacity score on ORACLe, albeit still in the moderate range (Fig 2). This score was reflective of the agency’s reportedly limited to moderate skill-building opportunities for leaders and staff, limited resources to access research (i.e., lack of research dissemination across the agency or a comprehensive library), and unestablished methods to evaluate policies. In SAGE interviews, A6 policymakers reported limited efforts to search for research, and to appraise its quality and relevance (Fig 3).

A2 and A4 displayed inconsistent ORACLe and SAGE profiles. A2 had the highest overall capacity to support research use in policy according to their ORACLE score (Fig 2). This agency reported extensive mechanisms to build leaders’ capacity to support research use (training programs and communications from leaders to staff) and to establish relationships with researchers, as well as training programs for staff that were incorporated into performance management processes. They also reported having undertaken internal and external research projects in the past six months. In terms of actual research use, however, SAGE data showed limited-moderate efforts to search for, and appraise the quality and relevance of research, and A2 obtained a low-moderate range of research (Fig 3). Furthermore, instrumental research use was in the limited-moderate range for A2, and imposed use was limited (Fig 4).

A4, in contrast, scored in the moderate range for capacity to support research use and achieved the lowest ORACLe score amongst our agencies (Fig 2). Although having some resources to access research (e.g., frequent research dissemination; journal and database access, and methods to commission reviews) and multiple mechanisms to establish relationships with researchers, this agency lacked clear processes for developing evidence-based policies, limited tools to support leaders, an absence of staff training programs, and limited documented processes to evaluate policies. In contrast, SAGE data showed that A4 policymakers’ exhibited extensive instrumental and tactical research use, and moderate-extensive conceptual use (Fig 4). Further, A4 policymakers’ efforts to generate research and interact with researchers were also extensive (Fig 3).

## Discussion

This is the first study to examine the extent to which Australian health agencies have tools and systems in place to support the use of research in policymaking. Having identified which tools and systems were present in six agencies we then explored their relationship with policymakers’ actual use of research in the development of policy documents. The results showed that, in general, agency executives reported a moderate to high level of organisational capacity to support their staff in using research in policymaking. There were both commonalities and differences between agencies in which tools and systems were available, shedding light on areas for potential improvement. In general, where agencies had extensive tools and systems in place to support research use, policymakers in those agencies displayed moderate to extensive efforts to engage with and/or use research in policymaking. However, two agencies displayed an inversion of research use capacity and actual engagement/use which indicates other mechanisms at work. In the discussion below we discuss the capacity domains that were particularly well developed across agencies, as well as those domains which could be further strengthened. We then discuss the relationship between these capacity domains and policymakers’ research engagement and use in policymaking and the implications for our understanding of the relationship between organisational capacity and research use.

### Agencies’ capacity to support research use in policymaking

All agencies reported at least a moderate level of capacity to support research use, with four displaying extensive overall capacity. Most agencies had a range of tools and systems to help staff access and apply existing research to policy, such as access to databases, libraries, reference management software, and research expertise; methods for commissioning research reviews; and frequent organisation-wide dissemination of research. In a previous study, this domain emerged as one of the most important areas of research use capacity [34]. Furthermore, numerous studies have raised the importance of developing such technical infrastructure to access existing research findings [17, 19, 43–45]. In support, among the three agencies that had the most extensive availability of tools and supports to access existing research (A3-A5), policymakers’ average engagement with research was in the moderate to extensive range.

All agencies reported having a number of processes to establish relationships with researchers. Studies show that sustained partnerships between researchers and policymakers encourage greater use of research in policymaking, for example, by informing priorities and recommendations, increasing the intention to use research, improving researchers’ understanding of the policy context, and increasing researchers’ capacity to develop research products suitable for policymakers (e.g., evidence briefs) [20, 58, 59]. Quantitative research has verified these findings, demonstrating significant associations between the intensity of policymaker-researcher linkages and eventual research use in policy [15]. In the present study, policymakers from agencies that reported extensive processes to establish relationships with researchers also displayed moderate to extensive research engagement actions and/or research use when developing policies (except A2).

One of the advantages of establishing relationships with researchers is coproduction of research to inform policy. Evidence suggests that policymakers are more likely to use research if they are jointly involved in the project as integral partners [4, 19–21, 36, 38], and our results are generally consistent with this perspective. Only half the agencies in the present study, however, commissioned an external research institute to conduct research to inform its policies in the past six months. Rather, most conducted internal research, often carried out by research units within the agency or the policy team themselves. This suggests that most agencies had systems for conducting internal research, although mechanisms to facilitate coproduction with external researchers could be developed further, as well as mechanisms to encourage ongoing relationships with researchers beyond the life of individual research projects. Ongoing policymaker-researcher relationships are vital to ensure researchers are aware of pressing policy issues so they can produce relevant and timely research [60]. To facilitate the formation of ongoing relationships, numerous courses have been developed, particularly in the USA and Canada, that aim to increase researchers’ understanding of knowledge translation and exchange (KTE) theory and concepts, improve their skills in developing KTE strategies and embedding these into research projects, enhance their capacity to build relationships with policymakers, and improve their ability to communicate research findings clearly and accessibly, to effectively engage policymakers [61–67].

A number of key areas of research use capacity could potentially be improved across agencies. The first of these are programs targeting agency leaders (domain 2). In our previous study, this domain was rated as the second most important for supporting organisational capacity to use research [68]. Such leadership-targeted programs are essential given that leaders are critical to establishing an evidence-based organisational culture [19, 42, 69], including the promotion of mutual understanding and collaboration between policymakers and researchers [18, 42], and advancing evidence-informed policy initiatives [42, 69]. Without a culture that supports evidence-informed policy it is unlikely that staff will value research, and use it to inform policy development [6, 11, 20].

In terms of additional resources to access existing research findings, studies indicate that policymakers may also benefit from being provided with efficient ways of storing, sharing, and retrieving knowledge from research [6, 19, 24, 45], and standardised frameworks on how to access and apply research to policy [18, 43]. These resources were generally lacking across our agencies, suggesting another potential area for improvement.

Although agencies generally appeared to value research use, the results suggested this could be made more explicit in relation to the policymaking process. For example, although four agencies had documentation on how to develop policy, only one (i.e., A1) had documentation instructing policymakers to consult the latest research. Interestingly, this was the only agency that displayed extensive instrumental, tactical, and conceptual research use, perhaps indicating the importance of having policy frameworks that explicitly encourage staff to seek out a broad range of evidence, including research. Furthermore, although five agencies required evaluation to be built into policy development, only one agency had a documented process for how policies should be evaluated, which was neither based on research nor instructed staff to seek out research to inform the evaluation design. It would be difficult, however, for most agencies to develop a standardised protocol for evaluating the wide variety of policies they develop. A feasible alternative would be for agencies to develop flexible evaluation guidelines that could be adapted to different policies and programs, and which advise staff to seek out relevant research to inform evaluation protocols.

Another area for potential development across most agencies was training programs for staff (domain 3). One agency (A3) provided these programs actively and regularly, two offered such programs on a needs basis, whereas three agencies did not provide any training. Policymakers have raised the importance of opportunities for training to improve their skills in accessing, appraising and applying research to policy [6, 44]. The availability of tools (e.g., database access, knowledge management systems) extends organisational capacity only if staff know how to use them. Indeed, studies have found that provision of training by the organisation is strongly associated with research use [24]. In support, two of the three agencies that provided staff with access to training (A2, A5) also scored in the moderate-extensive range for research engagement actions and use, reinforcing the importance of developing this capacity.

### Relationship between organisational capacity and policymakers’ engagement with and use of research

A unique feature of this study was our examination of the relationship between agencies’ research use capacity and policymakers’ actual research use in the development of policy documents. Results indicated that agency capacity, as measured by the tools and systems in place to support research use within the agency, predicted policymakers’ engagement with, and use of research in policy development for four of the six agencies.

A1, A3, and A5 each scored in the extensive range for ORACLe, indicating a high level of capacity to support staff engagement with, and use of, research in policy. These agencies in particular had clear and documented processes to develop evidence-informed policies (and in A3 and A5, there were clear methods to evaluate these policies). They also provided staff with an extensive range of resources to access existing research (e.g., journal subscriptions, mechanisms to commission reviews, and access to research experts and librarians), and had multiple processes to establish relationships with researchers. Consistent with their level of capacity, staff in these agencies exhibited moderate to extensive efforts to engage with research during the policymaking process. In particular, staff in A1 and A3, were likely to undertake thorough searches for literature (e.g., examining academic databases, sources of authoritative grey literature) and find a moderate to extensive range of up-to-date research (e.g., peer-reviewed articles, systematic reviews, policies and data from external agencies). Furthermore, policymakers’ *use* of this research during policy development in these agencies was moderate to extensive overall. Specifically, conceptual use of research was extensive, particularly within A3; while instrumental and tactical use was extensive within A1, and moderate within A3 and A5.

In contrast, A6 displayed a moderate level of organisational research use capacity, although this agency had numerous domains of capacity in the limited range including limited access to training programs for staff and leaders, limited to moderate tools to access existing research and limited processes to evaluate policies. Their engagement with research was in the moderate range, but their efforts to search for research and appraise its relevance and quality were limited (and lower than the other agencies studied). Instrumental and tactical research use were both in the moderate range, and conceptual use was extensive, suggesting that this agency’s moderate level of capacity more strongly influenced how policymakers engaged with research (e.g., their efforts to search for and appraise research) than how they used it.

Two agencies displayed inconsistencies between their ORACLe profile and their staff’s engagement with and use of research, as measured using SAGE. A2 showed extensive capacity to support research use, however, policymakers’ efforts to search for, appraise, and generate research were either limited or in the limited-moderate range. Their overall research engagement actions and research use scores were also in the limited to moderate range. In contrast, A4 displayed a moderate overall level of capacity. However, SAGE data showed A4 policymakers’ research engagement actions were on average in the moderate-extensive range. Furthermore, their use of research, on average, was in the extensive range.

The lack of relationship between capacity and staff research engagement and use for these two agencies indicates the influence of other factors on policymakers’ research-related behaviour not directly captured in ORACLe. According to the SPIRIT Action Framework (see Fig 1), besides the presence of systems and tools to support research use, an equally important component of capacity is an organisational *culture* where research use is valued by both staff and the organisation. Implementation research has shown that organisations with cultures that support evidence-based practices (EBPs), encourage innovation, emphasise competence and maintaining up-to-date knowledge, value staff input into decision making, and exhibit greater use and sustainability of new EBPs across the organisation [70–75]. ORACLe does not measure key aspects of culture, such as leadership, behavioural norms and collective expectations among staff regarding how to undertake their work [70–74], or the underlying assumptions and values that influence these behavioural expectations [70–74]. It is possible that these unmeasured components drove the discrepancies that were observed for agencies 2 and 4. For example, research use may not have been valued or expected in the organisational culture of A2, possibly due to leadership characteristics or contextual factors unique to this agency and its work [74]. In contrast, the relatively new executive in A4 may have strongly valued research, but this was not captured in their ORACLe scores because their tools and systems use were still in development and had yet to ‘catch up’ with the agency’s emerging research culture.

These findings suggest that the availability of tools and systems within the organisation is generally predictive of policymakers’ engagement with and use of research in policy development. This may be contingent, however, upon the organisation having an underlying culture where research use in policy is valued.

The results indicate that capacity influences not only instrumental and conceptual uses, but also greater tactical use. Organisations with high scores on capacity generally scored in the moderate to extensive range on tactical research use (particularly A3). The SPIRIT Action framework proposes that research-*informed* policies may lead to improvements in health services and outcomes. It can be argued that research *informs* policy only when it is used instrumentally and/or conceptually. For both types of use, research comes *before* a decision is made, and is used to help solve important policy-related issues, consistent with the problem-solving model of research use proposed by Weiss [31]. Tactical use, on the other hand, comes *after* a policy decision, where research is used to justify decisions already made. Consequently, research findings may be accessed or reported selectively in order to support these decisions, implying that such tactical use should be discouraged. The reality, however, is that policymaking is highly political [33, 76]. Decisions are often made in response to political pressures or stakeholder interests and consultation, before research evidence has been considered. Research is, therefore, often used tactically as a means of addressing, responding to, or influencing these stakeholders [76, 77]. To the extent that research is used in a *non-biased* fashion to present the complete facts and most effective strategies to stakeholders, promote discussion and collaboration, and counteract potentially ineffective courses of action, tactical research use may elicit positive outcomes. However, tactical use involving selective and biased searching and reporting of findings to justify positions, represents a misuse of research and should therefore not be encouraged. Policy agencies aiming to promote a culture of research use may benefit from clarifying this distinction to staff, in order to promote unbiased research use in policy formulation.

### Strengths and limitations

This study had a number of strengths. Firstly, the data presented in this paper represents the first detailed and systematic investigation of the tools and systems available to support research use in policy within Australian agencies with a role in health policy. Secondly, we used newly developed, validated, and reliable instruments to measure both the capacity of organisations to support research use (i.e., ORACLe [34]) and policymakers’ actual engagement with and use of research in policy development (i.e., SAGE [53–56]). Third, and most important, we qualitatively evaluated the relationship between organisational capacity, and the extent to which policymakers actually engaged with, and used research during the policy development process. These findings provide valuable insights regarding the importance of building organisational capacity, and shed light on which tools and systems could be developed further to enhance organisations’ overall capacity to support research use, and thereby improve how policymakers engage with, and use research in policymaking.

The study also had some limitations. Firstly, our inferences regarding the relationship between culture, capacity, and research use behaviour are speculative since we did not measure (i) organisational culture from the staff perspective, or (ii) the agency’s underlying assumptions and values towards research use. Staff perceptions of culture could be assessed with the validated Organisational Social Context measure [70, 71], which evaluates organisational culture, climate, and staff work attitudes constructs in detail. Another recently instrument recently developed by CIPHER that could also be utilised in future research is SEER (Seeking, Engaging, Evaluating Research), which includes subscales assessing policymakers’ attitudes, and perceptions of the organisation’s attitudes, towards research use in policy. These SEER subscales have demonstrated good test-retest reliability and construct validity [78].

Measurement of underlying assumptions and values regarding research use, is more difficult as these are less consciously accessible. According to Glisson and James [73] this knowledge may be obtained indirectly by examining the behaviour and statements of individuals in the organisation, for example, through in-depth ethnographic studies of policymakers at work. However, conducting this type of research is highly intensive and may be impractical or unwelcome in many policy contexts.

Secondly, our analysis is based primarily on observational data from a small number of agencies based in Sydney, Australia. In order to make more definitive conclusions regarding the relationship between capacity and research use, future studies could recruit a large number of agencies and utilise multilevel modelling to determine whether organisational capacity and culture interacts with staff capacity to predict policymakers’ research engagement actions and research use during the policy development process.

### Conclusion

In this study, we have documented and quantified the tools and systems to support research use within six Australian agencies with a role in health policy, and examined their relationship to policymakers’ use of research in policy. The results showed that overall, all agencies possessed at least a moderate level of capacity to support research use. Capacity domains that were well developed included the presence of tools to access existing research (e.g., databases and journal access), mechanisms to develop relationships with researchers, and systems to generate research to inform policy. Areas requiring further development included training programs for leaders and other staff, and processes for conducting research-informed evaluations of policies. For most agencies, the availability of tools and systems predicted better engagement with, and use of research in policymaking. In two agencies, however, this relationship did not emerge, implying that other contextual factors such as organisational culture should be taken into account when attempting to improve policymakers’ engagement with and use of research. The present study provides insights into the importance of organisations building capacity to support the use of research, and highlights particular tools and systems that can contribute to this capacity.
